# Hydrogen improves neurological function through attenuation of blood–brain barrier disruption in spontaneously hypertensive stroke-prone rats

**DOI:** 10.1186/s12868-015-0165-3

**Published:** 2015-04-20

**Authors:** Satoru Takeuchi, Kimihiro Nagatani, Naoki Otani, Hiroshi Nawashiro, Takashi Sugawara, Kojiro Wada, Kentaro Mori

**Affiliations:** Department of Neurosurgery, National Defense Medical College, 3-2 Namiki, Tokorozawa, Saitama 359-8513 Japan; Division of Neurosurgery, Tokorozawa Central Hospital, Tokorozawa, Saitama Japan; Department of Neurosurgery, Tokyo Medical and Dental University, Tokyo, Japan

**Keywords:** Hydrogen-rich water, Oxidative stress, Blood–brain barrier, Spontaneously hypertensive stroke-prone rats

## Abstract

**Background:**

Enhanced oxidative stress occurs in spontaneously hypertensive stroke-prone rats (SHRSP), and is important in blood–brain barrier (BBB) disruption. Hydrogen can exert potent protective cellular effects via reduction in oxidative stress in various diseases. The present study investigated whether long-term hydrogen treatment can improve neurological function outcome in the SHRSP model, and the effects of hydrogen on BBB function, especially the oxidative stress and the activity of matrix metalloproteinases (MMPs) in this model. Fifty-six animals were randomly assigned to 2 groups and treated as follows: SHRSP treated with hydrogen-rich water (HRW) (HRW group, *n* = 28); and SHRSP treated with regular water (control group, *n* = 28). The effect of HRW on overall survival and neurological function, and the effects of HRW on reactive oxygen species, BBB function, and MMP activities were examined.

**Results:**

HRW treatment improved neurological function and tended to improve overall survival but without significant difference. The numbers of bleeds and infarcts were lower in the cortex and hippocampus in the HRW group. The HRW group exhibited a significantly lower number of 8-hydroxy-2'-deoxyguanosine-positive cells and vessels of extravasated albumin in the hippocampus compared with the control group. MMP-9 activity was reduced in the hippocampus in the HRW group compared with the control group.

**Conclusions:**

The present study suggests that ingestion of HRW can improve neurological function outcome in the SHRSP model. This beneficial effect may be due to attenuation of BBB disruption via reduction in reactive oxygen species and suppression of MMP-9 activity in the hippocampus.

## Background

Spontaneously hypertensive stroke-prone rats (SHRSP), a substrain of spontaneously hypertensive rats (SHR) derived from Wistar Kyoto rats, develop malignant hypertension at an early age and have an increased incidence of spontaneous hemorrhagic and ischemic strokes [1-3]. In this model, cerebral hemorrhage or infarction occurs in 82% of males over 100 days of age and 58% of females over 150 days of age [1]. Therefore, SHRSP has been used as a suitable experimental model for the investigation of hypertension-related cerebrovascular diseases such as cerebral hemorrhage and infarction [1-3]. There is increasing evidence suggesting that blood–brain barrier (BBB) disruption is the key factor for the onset of stroke in SHRSP [4-6], and several studies have shown that BBB disruption preceded strokes in SHRSP [5-7]. Increased vascular permeability preceded intracerebral hemorrhage in SHRSP using magnetic resonance imaging [5]. Furthermore, stroke originated from BBB disruption in SHRSP based on microscopy studies [6]. BBB disruption has been shown to start from 12–15 weeks of age in SHRSP [5,6]. BBB disruption occurs in several regions including the cortex, hippocampus, basal ganglia, and corpus callosum in SHRSP, and the extent of BBB disruption increases age-dependently. In contrast, the most localized BBB disruption was clearly seen in the hippocampus in SHRSP aged 3 months [7]. Furthermore, BBB disruption in SHRSP is considered to be the result of enhanced oxidative stress [8-12].

Matrix metalloproteinases (MMPs), members of the family of zinc-dependent endopeptidases, are fundamental in extracellular matrix physiology [13]. Two specific MMPs, MMP-2 (gelatinase A) and MMP-9 (gelatinase B), have been the subjects of extensive studies in the stroke field. Activated MMPs have been reported in SHR and SHRSP [14]. MMPs, in particular MMP-9, are another important factor in BBB disruption [15-18]. Furthermore, several studies showed that MMP activities may be regulated by reactive oxygen species (ROS) [19-21].

Hydroxyl radicals and peroxynitrites are very strong ROS that react indiscriminately with nucleic acids, lipids, and proteins, resulting in DNA fragmentation, lipid peroxidation, and protein inactivation [22]. Since hydrogen gas was reported to reduce infarct size and improve neurological function in the rat middle cerebral artery occlusion model by selectively scavenging hydroxyl radicals and peroxynitrites [23], the effects of hydrogen have been studied extensively on several pathologies in the central nervous system, heart, kidney, liver, and intestines [21-31]. Hydrogen has been shown to have potent protective cellular effects by reduction of oxidative stress markers, including 8-hydroxy-2'-deoxyguanosine (8-OHdG) (nucleic acid oxidation marker), 4-hydroxynonenal (4-HNE) (lipid peroxidation marker), and malondialdehyde (lipid peroxidation marker) [21-31]. In particular, intraperitoneal hydrogen-rich saline injection reduced infarct volume and improved neurological function via attenuation of neuronal injury in a rat common carotid artery occlusion and hypoxia model [30], and hydrogen gas improved survival rate via attenuation of neuronal injury, autophagy, and BBB disruption in a mouse bilateral common carotid artery occlusion model [25]. Furthermore, intrathecal hydrogen-rich saline injection improved neuropathic pain behavior by reducing ROS in the spinal astrocytes and microglia in a rat neuropathic model [31].

Hydrogen therapy is usually administered by three methods, inhalation of hydrogen gas, injection of hydrogen-rich saline, and ingestion of hydrogen-rich water (HRW) [21-30]. Ingestion of HRW is considered to be safer and more convenient and suitable for long-term treatment [21]. Hydrogen can downregulate many molecules, including MMP-9 and/or MMP-2 [27,28]. Hydrogen gas decreased oxidative stress (8-OHdG, 4-HNE, and nitrotyrosine) and exerted neuroprotective effects (improvement of neurological score and reduction in infarct and hemorrhagic volumes), and also reduced MMP-9 activation in a rat middle cerebral artery occlusion model [28]. However, whether long-term hydrogen treatment can improve neurological function under chronic hypertensive conditions is unknown.

The present study investigated (1) whether long-term (8–16 weeks) HRW treatment can improve outcome in the SHRSP model, and (2) the effect of HRW on BBB function, focusing on the oxidative stress and the activity of MMPs at the early stage in the SHRSP model.

## Methods

### Animals and experimental procedures

All experimental procedures were approved by the Animal Care and Use Committee of the National Defense Medical College. Fifty-six male SHRSP at 6 weeks of age were purchased from Japan SLC (Shizuoka, Japan). The rats were housed in individual cages under controlled environmental conditions (12/12 h light/dark cycle, room temperature at 20–22°C) with ad libitum access to Funabashi SP diet, which contains 19.7% crude protein, 4.8% crude fat, 3.4% crude fiber, and 3.7 mg/g of sodium (Funabashi Farm, Chiba, Japan), and water. Animals at 6 weeks were randomly assigned to 2 groups and treated as follows: (1) SHRSP treated with HRW (HRW group, *n* = 28); and (2) SHRSP treated with regular water (control group, *n* = 28). Body weight was measured every day. Blood pressure and heart rate were measured by the tail cuff method in conscious rats, using a non-preheating, non-invasive blood pressure monitor (MK-2000ST, Muromachi Kikai, Tokyo, Japan) weekly. Twenty animals were observed until 16 weeks after treatment (22 weeks of age) for survival analysis (*n* = 10 in each group). Surviving animals at 16 weeks after treatment were sacrificed for histological examination with hematoxylin and eosin (HE) staining. In addition, 36 animals at 8 weeks after treatment (14 weeks of age) were sacrificed for histological examination with HE and immunohistochemistry (*n* = 6 in each group), gelatin zymography (*n* = 6 in each group), or serum 8-OHdG analysis (*n* = 6 in each group). All assays and measurements in this study were performed by investigators unaware of the experimental groups.

### Preparation of HRW

HRW was made using hydrogen water 7.0 (Ecomo International Co., Ltd., Fukuoka, Japan), as previously reported [26]. Briefly, hydrogen gas was produced in an acrylic resin tube and introduced into a polyethylene terephthalate (PET) bottle. The PET bottle was completely filled with water. Hydrogen gas was produced from 0.5 g of 75% by weight of metal aluminum grains and 25% by weight of calcium hydroxide. This material with 0.5 mL of water was inserted into an acrylic resin tube, and a cap with a check valve was tightly closed. After 5 minutes, the reaction started in the wet fabric. The hydrogen gas produced was passed through the check valve into the water in the PET bottle. After the hydrogen-generating reaction was terminated, the hydrogen gas was dissolved by shaking the bottle. The concentration of hydrogen in water was measured by titration using methylene blue-platinum colloid reagent as previously reported [29], and the measured concentration was 5 ppm.

### Oral administration of HRW

HRW was prepared each day and 200 mL per rat was delivered twice a day at 20-22°C in glass bottles (200 mL) with tight rubber caps to rats in the HRW group. Regular water was given to rats in the control group. The water temperature was monitored and maintained at 20-22°C.

### Monitoring of neurological deficits and death

The appearance of neurological deficits was carefully monitored every day. The neurological deficits were evaluated according to a scoring system: 0, normal; 1, slight decrease in motor activity or slight excitement; 2, marked decrease in motor activity or hyperirritability; 3, no walking (decreased responsiveness); 4, inability to stand without support or paralysis of hind limbs; and 5, death [32]. Macroscopic autopsy of the brain, lung, heart, aorta, bilateral carotid arteries, liver, kidney, and intestine was performed on dead animals to determine the cause of death.

### Blood collection

Blood samples were collected transcardially under anesthesia induced with an overdose of pentobarbital sodium (100 mg/kg) by intra-peritoneal injection at 8 weeks after treatment. Serum samples were obtained after centrifuging for 15 minutes at 3,000 rpm, and used for the assay of serum 8-OHdG.

### Tissue preparation

For histological examination with HE and immunohistochemical studies, animals were perfused transcardially with normal saline, followed by 4% buffered paraformaldehyde under anesthesia induced with an overdose of pentobarbital sodium by intra-peritoneal injection at 8 or 16 weeks after treatment. The brain was removed and embedded in paraffin after fixation in 4% buffered paraformaldehyde, followed by 0.1 mmol/L phosphate-buffered saline (pH 7.4) for 24 hours at 4°C. The 5-μm coronal sections were prepared. For the gelatin zymography study, animals were decapitated under anesthesia induced with an overdose of pentobarbital sodium by intra-peritoneal injection and their brains were removed, quickly cut into several regions including the cortex and the hippocampus.

### Measurement of serum 8-OHdG

Serum samples obtained from blood samples were used for the measurement of 8-OHdG. Serum 8-OHdG was determined by enzyme-linked immunosorbent assay following the manufacturer’s instructions (Highly Sensitive 8-OHdG Check, Institute for the Control of Aging, Fukuroi, Shizuoka, Japan). Optical density was measured at 450 nm using a microplate reader (Microplate EIA Autoanalyzer AP-960 system, Kyowa Medex, Tokyo, Japan). Quantification of 8-OHdG was achieved by comparing the optical densities of each sample to that of internal standards of various known concentrations of 8-OHdG.

### HE staining

Ten sections per animal (from the frontal to the occipital pole) were prepared. One slice per section was stained with HE for surviving animals at 16 weeks after treatment (control group *n* = 3, HRW group *n* = 6) and animals at 8 weeks after treatment (*n* = 6 per group). Ten slices per animal were observed to count the numbers of bleeds and thromboses/infarcts. Sections were examined using a microscope (Axio Imager.A1, Carl Zeiss, Oberkochen, Germany) equipped with a digital camera system (Axio Cam MRc 5, Carl Zeiss).

### Immunohistochemistry

The sections (anteroposterior coordinate, bregma −3.8 mm) from animals sacrificed at 8 weeks after treatment were stained overnight at 4°C with anti-8-OHdG (mouse monoclonal, Japan Institute for the Control of Aging, Shizuoka, Japan; 1:200) and anti-albumin (sheep polyclonal, Bethyl Laboratories, Inc., Montgomery, TX; 1:500). Sections were covered with mounting medium containing 4,6-diamidino-2-phenylindole (DAPI) for fluorescence microscopy (Vector Laboratories, Burlingame, CA). Immunoreactivity was detected using a diaminobenzidine method. For quantitative analysis, the number of 8-OHdG-positive cells and the number of vessels with extravasated albumin signals were counted in the bilateral cortices and hippocampi [25,33]. Six slices (including cortex and hippocampus) per animal were evaluated, and the average count per high power field of view was calculated from the bilateral cortices and hippocampi, respectively.

For double staining, the sections were incubated in a nonspecific blocking reagent (Dako, Glostrup, Denmark) for 30 minutes to reduce background staining. Sections were then incubated with primary antibodies overnight in a humidified chamber at 4°C. Primary antibodies were anti-8-OHdG (Japan Institute for the Control of Aging; 1:200), anti-MMP-2 and −9 (mouse monoclonal, Novus Biologicals, Littleton, CO; 1:100), anti-neuronal nuclear antigen (NeuN) (neuronal marker) (rabbit polyclonal, Millipore, Billerica, MA; 1:200), anti-glial fibrillary acidic protein (GFAP) (astrocyte marker) (rabbit polyclonal, Thermo Fisher Scientific, Waltham, MA; 1:200), and anti-Iba-1 (microglia marker) (rabbit polyclonal, Wako, Osaka, Japan; 1:200) antibodies. For fluorescent staining, sections were incubated with Alexa-Fluor 488-conjugated goat anti-rabbit immunoglobulin G (Molecular Probes, Eugene, OR; 1:200) for primary antibodies derived from the mouse. For primary antibody derived from the rabbit, Alexa-Fluor 546-conjugated goat anti-mouse immunoglobulin G (Molecular Probes; 1:200) was used. Sections were examined using a microscope (Axio Imager.A1) equipped with a digital camera system (Axio Cam MRc 5).

### Gelatin zymography

Metalloproteinase extraction from brains was performed according to a previously described method [34]. Briefly, the frozen tissue samples were homogenized with a lysis buffer (50 mmol/L Tris–HCl [pH 7.4], 5 mmol/L CaCl_2_, 1 μmol/L ZnCl_2_, and 0.05% BRIJ-35). The homogenates were centrifuged at 12,000 *g* for 15 minutes. The supernatants were recovered and total protein concentrations were measured (BCA kit, Pierce, Rockford, IL). Each lane was loaded with 20 μg total plasma protein with loading buffer (125 mmol/L Tris–HCl [pH 6.8], 4% sodium dodecyl sulfate [SDS], and 10% sucrose) at a 1:1 ratio. Gelatin at a concentration of 0.1% was incorporated into the 10% polyacrylamide gel containing 0.4% SDS. Electrophoresis was performed at 25 mA until the bromophenol blue marker dye reached the bottom of the gel. After washing in 2.5% Triton X-100 to remove SDS, the gels were incubated overnight in a developing buffer (50 mmol/L Tris–HCl [pH 7.4], 5 mmol/L CaCl_2_, 1 μmol/L ZnCl_2_, and 0.05% BRIJ-35) at 37°C. The gels were then stained with 0.1% Coomassie blue and destained. Densitometric analysis used the NIH ImageJ software program (http://rsb.info.nih.gov/ij/).

### Statistical analysis

The data are presented as means ± SEM. Survival rates were examined using the Kaplan-Meier method. The survival curves were compared using a log-rank test. Comparisons between two groups were performed with an unpaired *t*-test. A value of *P* < 0.05 was considered to be significant. The Graphpad Prism 6.0 software program (San Diego, CA) was used for all statistical tests.

## Results

### Physiological data

Age-related increases in body weight and blood pressure were observed in both groups, but the physiological data showed no significant differences between the control group and the HRW group (Table 1).

**Table 1:** **Physiological parameters before and 8 weeks after treatment**

	**BW (g)**		**SBP (mmHg)**		**DBP (mmHg)**		**HR (bpm)**	
	**Before**	**After**	**Before**	**After**	**Before**	**After**	**Before**	**After**
Control	101.8 ± 2.05	280.4 ± 6.56*	139.0 ± 3.65	241.7 ± 9.19*	79.3 ± 9.35	174.3 ± 4.85*	452.1 ± 14.15	466.8 ± 9.21
HRW	99.8 ± 1.91	288.5 ± 2.40*	137.3 ± 5.61	237.1 ± 10.4*	77.7 ± 5.74	168.4 ± 5.54*	464.3 ± 17.13	465.5 ± 5.29

BW: body weight; DBP: diastolic blood pressure; HR: heart rate; HRW: hydrogen-rich water; SBP: systolic blood pressure.

Data were analyzed by an unpaired *t*-test. Data are means ± SEM. *P < 0.05 vs before treatment; *n* = 10 in both groups.

### Neurological function and overall survival

**Figure 1:**
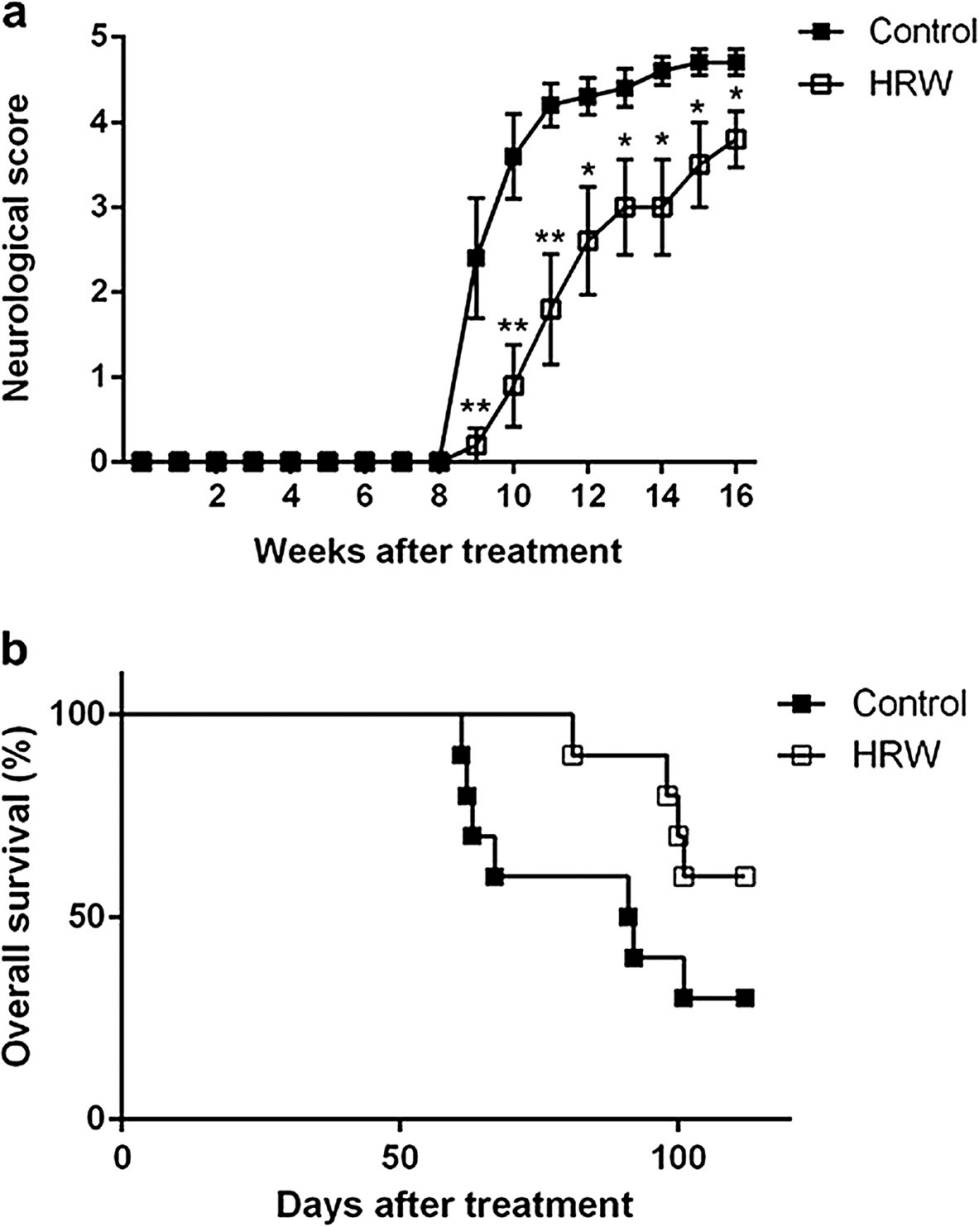
Effect of hydrogen-rich water (HRW) on neurological function and overall survival. **a**, neurological function; **b**, overall survival. Neurological score was higher in the control group than in the HRW group from 9 to 16 weeks after treatment. Overall survival tended to be longer in the HRW group (*P* = 0.094) but not significantly. Kaplan-Meier survival curves were analyzed by a log rank test. Neurological scores at each week were analyzed by an unpaired *t*-test. Values are expressed as mean ± SEM. *n* = 10 in each group. *P < 0.05, **P < 0.01 vs. control group.

**Figure 2:**
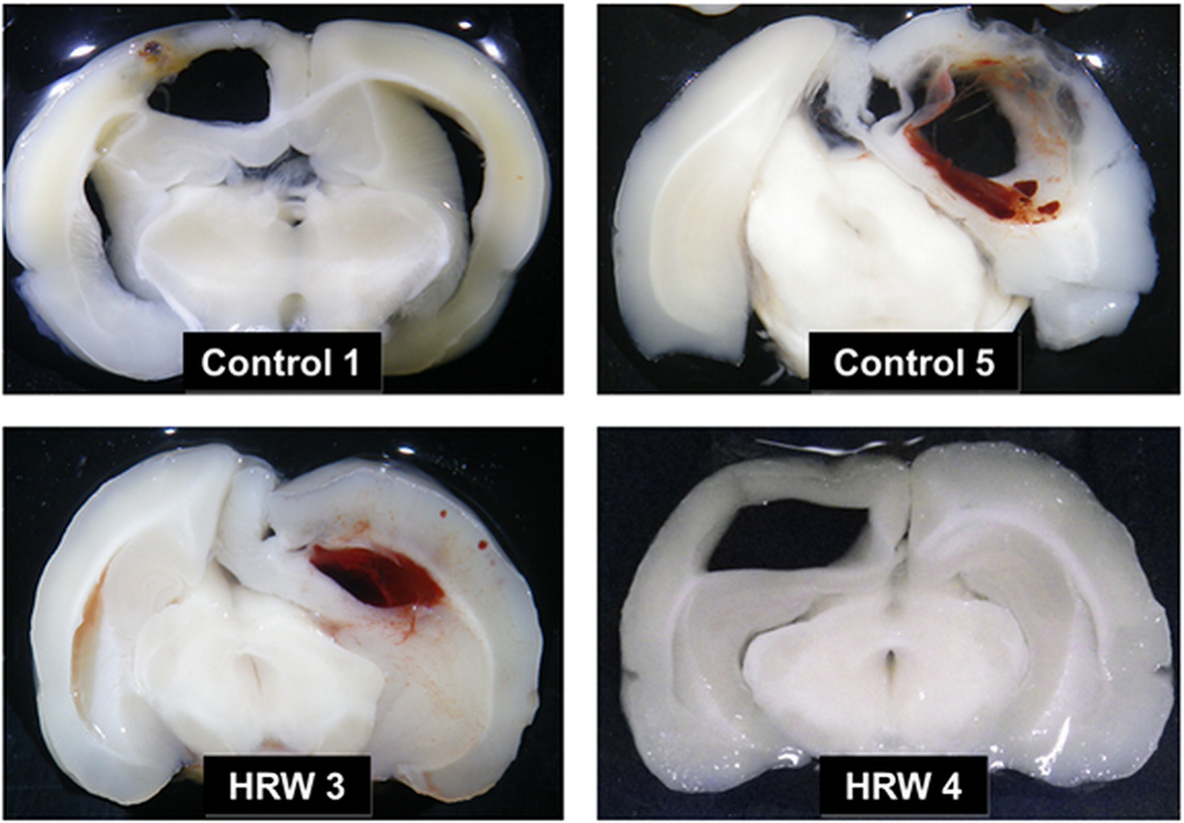
Macroscopic autopsy findings of dead animals. Representative photographs of brain autopsy in dead rats. Massive intracerebral hemorrhage was observed in all rats.

Neurological score was higher in the control group than in the HRW group from 9 to 16 weeks after treatment (Figure 1). In the control group, seven rats had died by 16 weeks after treatment. In the HRW group, four rats had died by 16 weeks after treatment. Autopsy showed that the cause of death was massive intracerebral hemorrhage in 9 rats (*n* = 6 in the control group, *n* = 3 in the HRW group) and sustained generalized seizure due to massive cortical hemorrhage in two rats (*n* = 1 per each group). No fatal lesions were found in the other organs (Table 2, Figure 2). The 16-week survival rate was 30% in the control group and 60% in the HRW group. Overall survival tended to show a difference (*P* = 0.094) but without significance (Figure 1).

**Table 2:** **Details of outcomes**

	**Outcome (days after treatment)**
Control 1	Death due to massive intracerebral hemorrhage (91)
Control 2	Survival
Control 3	Survival
Control 4	Survival
Control 5	Death due to massive intracerebral hemorrhage (63)
Control 6	Death due to massive intracerebral hemorrhage (101)
Control 7	Death due to massive intracerebral hemorrhage (92)
Control 8	Death due to generalized seizure caused by intracerebral hemorrhage (62)
Control 9	Death due to massive intracerebral hemorrhage (61)
Control 10	Death due to massive intracerebral hemorrhage (67)
HRW 1	Survival
HRW 2	Survival
HRW 3	Death due to massive intracerebral hemorrhage (98)
HRW 4	Death due to massive intracerebral hemorrhage (101)
HRW 5	Death due to generalized seizure caused by intracerebral hemorrhage (81)
HRW 6	Survival
HRW 7	Survival
HRW 8	Survival
HRW 9	Death due to massive intracerebral hemorrhage (100)
HRW 10	Survival

HRW: hydrogen-rich water.

### Effect of HRW on cerebral lesions

**Figure 3:**
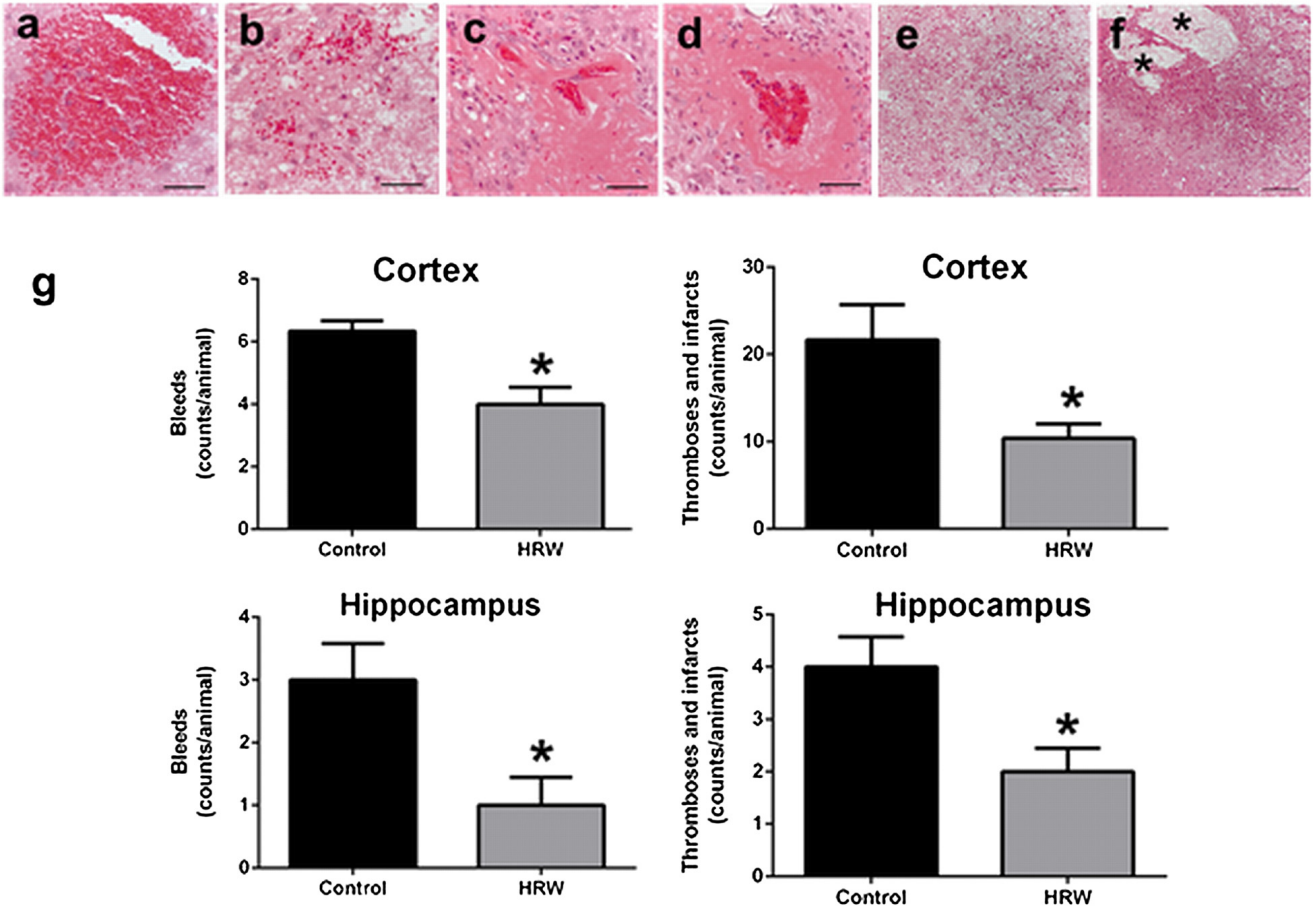
Effect of hydrogen-rich water (HRW) on cerebral lesions. Hematoxylin and eosin staining was performed in surviving animals at 16 weeks after treatment. Representative photographs showing bleeds in the cortex **(a)** and hippocampus **(b)**, thromboses in the cortex **(c)** and hippocampus **(d)**, and infarcts in the cortex **(e)** and hippocampus **(f)**. The infarcted areas included cells with vacuole-like structures and/or liquefaction (asterisks). Quantification of cerebral bleeds and thromboses/infarcts in the cortex and hippocampus **(g)**. The numbers of bleeds and thrombosis/infarcts were lower in the cortex and hippocampus in the HRW group. Data were analyzed by an unpaired *t*-test. Values are expressed as mean ± SEM. *n* = 3 in the control group, *n* = 6 in the HRW group. Scale bars = 50 μm **(a, b, c, d)** or 200 μm **(e, f)**. *P < 0.05 vs. control group.

No cerebral bleeds or thromboses/infarcts were observed in either group at 8 weeks after treatment. Numbers of bleeds and thromboses/infarcts were both significantly lower in the cortex and hippocampus in the HRW group at 16 weeks after treatment (*P* < 0.05) (Figure 3).

### Effect of HRW on serum 8-OHdG level

**Figure 4:**
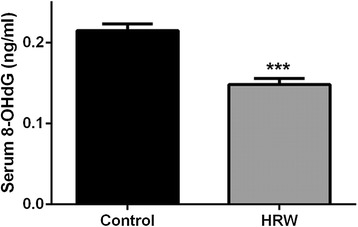
Effect of hydrogen-rich water (HRW) on oxidative DNA damage in the whole body. Oxidative DNA damage was analyzed using enzyme-linked immunosorbent assay of the serum 8-hydroxy-2'-deoxyguanosine (8-OHdG) level at 8 weeks after treatment. Serum 8-OHdG levels were significantly lower in the HRW group than in the control group. Data were analyzed by an unpaired *t*-test. Values are expressed as mean ± SEM. *n* = 6 in each group. ***P < 0.001 vs. control group.

Total oxidative stress in the rat body was assessed by measuring the serum 8-OHdG levels at 8 weeks after treatment. Serum 8-OHdG levels were significantly lower in the HRW group than in the control group (0.148 ng/mL vs 0.215 ng/mL, *P* < 0.001) (Figure 4).

### Effect of HRW on 8-OHdG immunoreactivity in the brain

**Figure 5:**
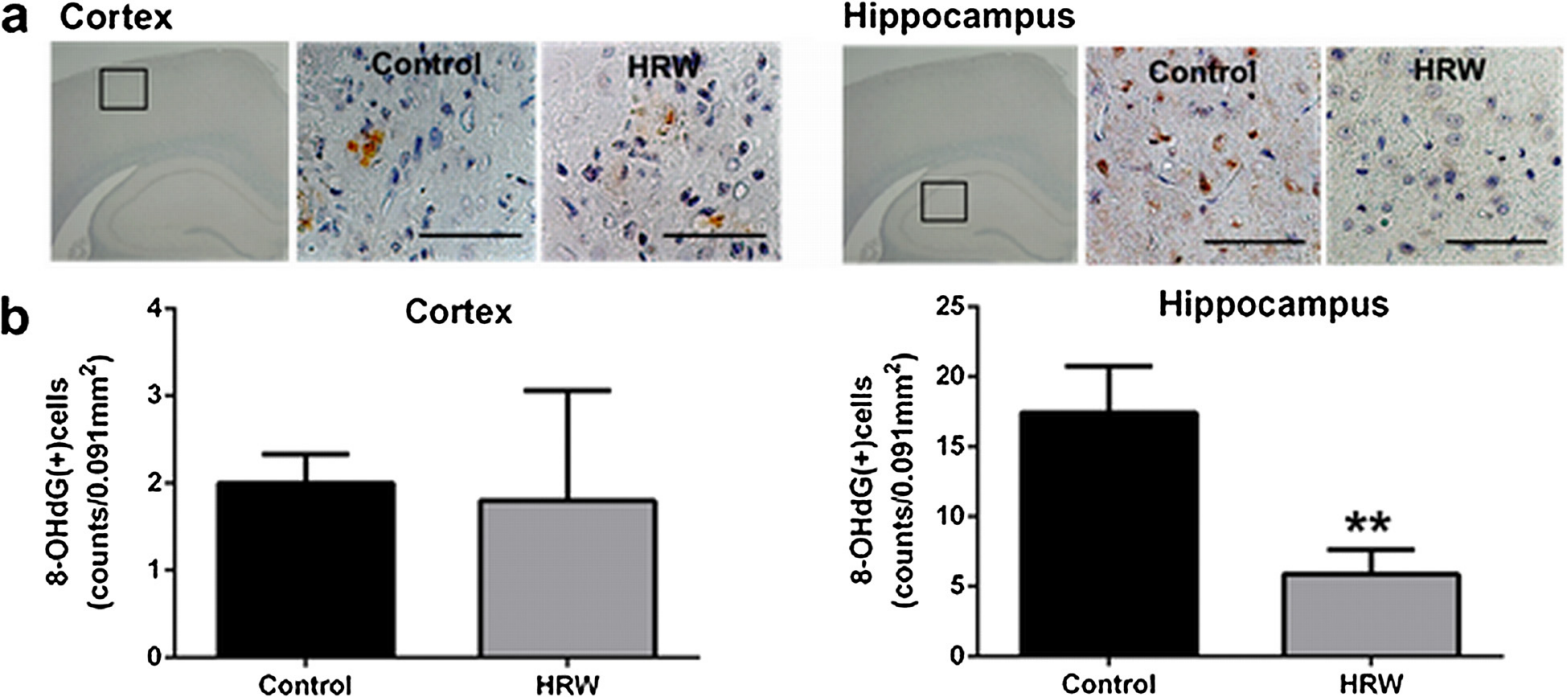
Effect of hydrogen-rich water (HRW) on oxidative DNA damage in the brain. **a**, Representative photomicrographs showing immunostaining for 8-hydroxy-2'-deoxyguanosine (8-OHdG). Each small box marks a region shown at higher magnification in an adjacent panel. **b**, Quantification of 8-OHdG-positive cells in the cortex and hippocampus. In the cortex, there was no significant difference in 8-OHdG immunoreactivity between the two groups (*P* = 0.77). In the hippocampus, the HRW group had significantly lower number of 8-OHdG-positive cells (*P* = 0.01). Data were analyzed by an unpaired *t*-test. Values are expressed as mean ± SEM. *n* = 6 in each group. Scale bars = 50 μm. **P = 0.01 vs. control group.

**Figure 6:**
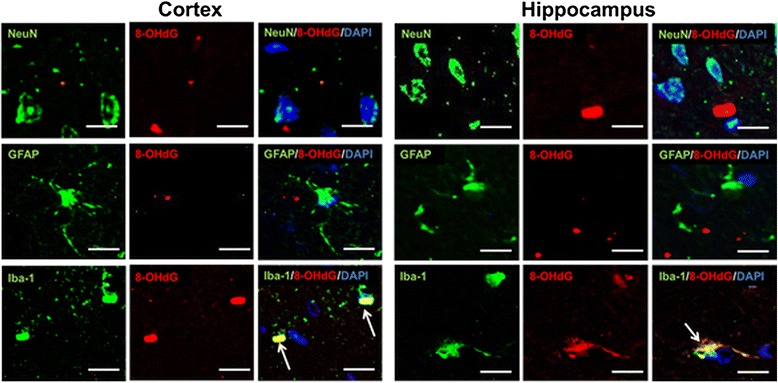
Immunofluorescence staining for 8-hydroxy-2'-deoxyguanosine (8-OHdG). Double staining for neuronal nuclear antigen (NeuN), glial fibrillary acidic protein (GFAP), and Iba-1 (green) showing that 8-OHdG (red) was expressed in the microglia (arrows), but not in neurons and astrocytes (nuclei, 4,6-diamidino-2-phenylindole [DAPI], blue). Co-localization is shown as yellow. Scale bars = 20 μm.

Oxidative DNA damage was assessed in the brain using an 8-OHdG antibody at 8 weeks after treatment. Faint 8-OHdG immunoreactivity was detected in the cortex in both groups, showing no significant difference (*P* = 0.77) (Figure 5). However, strong 8-OHdG immunoreactivity was observed in the hippocampus in the control group, with a significantly lower number of 8-OHdG-positive cells in the hippocampus in the HRW group (*P* = 0.01) (Figure 5). Double staining for NeuN, GFAP, and Iba-1 indicated that 8-OHdG was expressed in the microglia, but not in neurons and astrocytes (Figure 6).

### Effect of HRW on serum albumin extravasation

**Figure 7:**
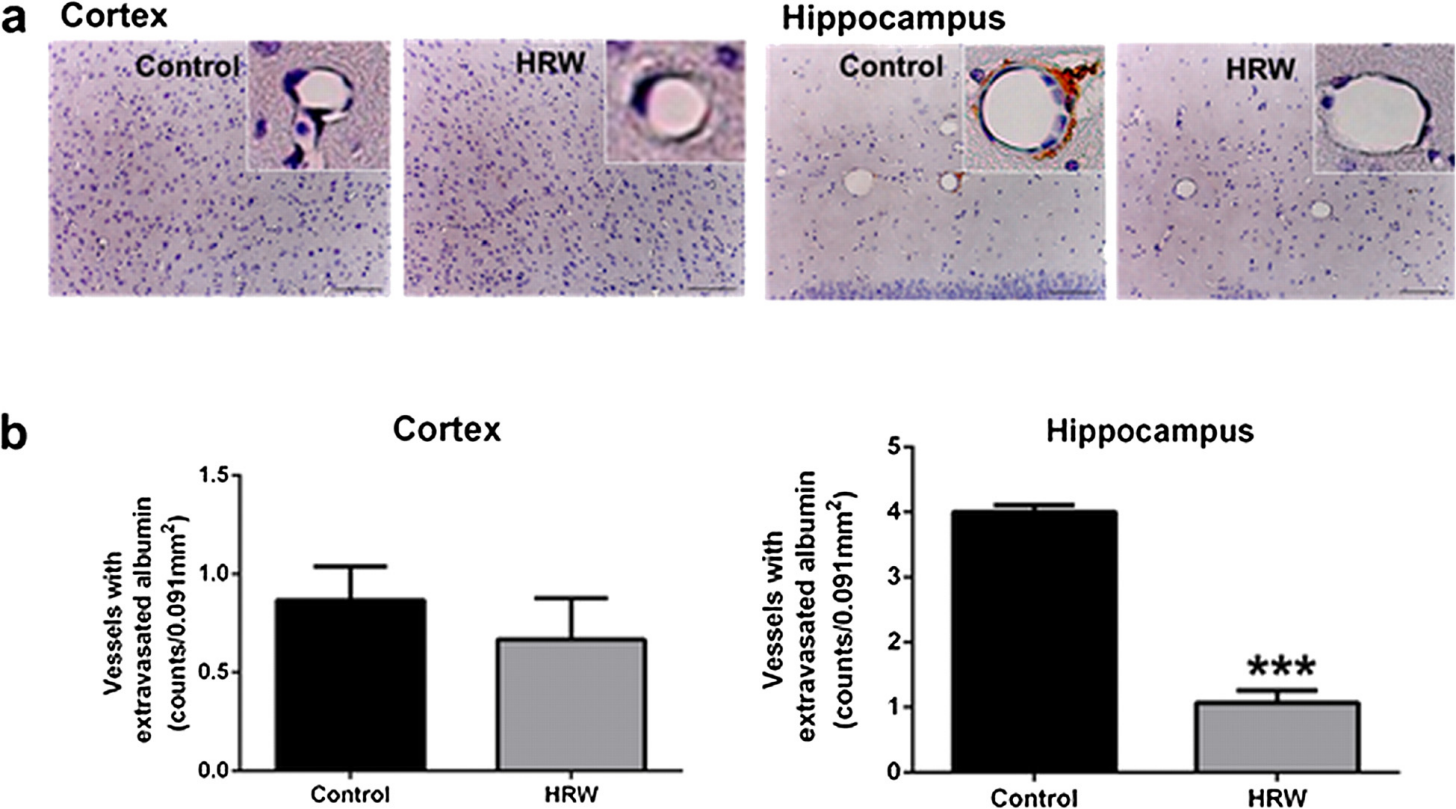
Effect of hydrogen-rich water (HRW) on the permeability of the blood–brain barrier. **a**, Representative photomicrographs showing immunostaining for albumin. **b**, Quantification of the number of vessels with extravasated albumin in the cortex and hippocampus. In the cortex, there was no significant difference in numbers of vessels of extravasated albumin between the two groups. In the hippocampus, the HRW group had a significantly lower number of vessels with extravasated albumin (*P* < 0.001). Data were analyzed by an unpaired *t*-test. Values are expressed as mean ± SEM. *n* = 6 in each group. Scale bars = 100 μm. ***P < 0.001 vs. control group.

Severity of BBB disruption was assessed by measurement of the number of vessels with extravasated albumin at 8 weeks after treatment. Faint albumin signals were detected in the cortex in both groups, with no significant difference (*P* = 0.48) (Figure 7). Strong extravasated albumin signals were detected in the hippocampus in the control group, with a significantly lower number of vessels with extravasated albumin in the hippocampus in the HRW group (*P* < 0.001) (Figure 7).

### Effect of HRW on MMP activity in the brain

**Figure 8:**
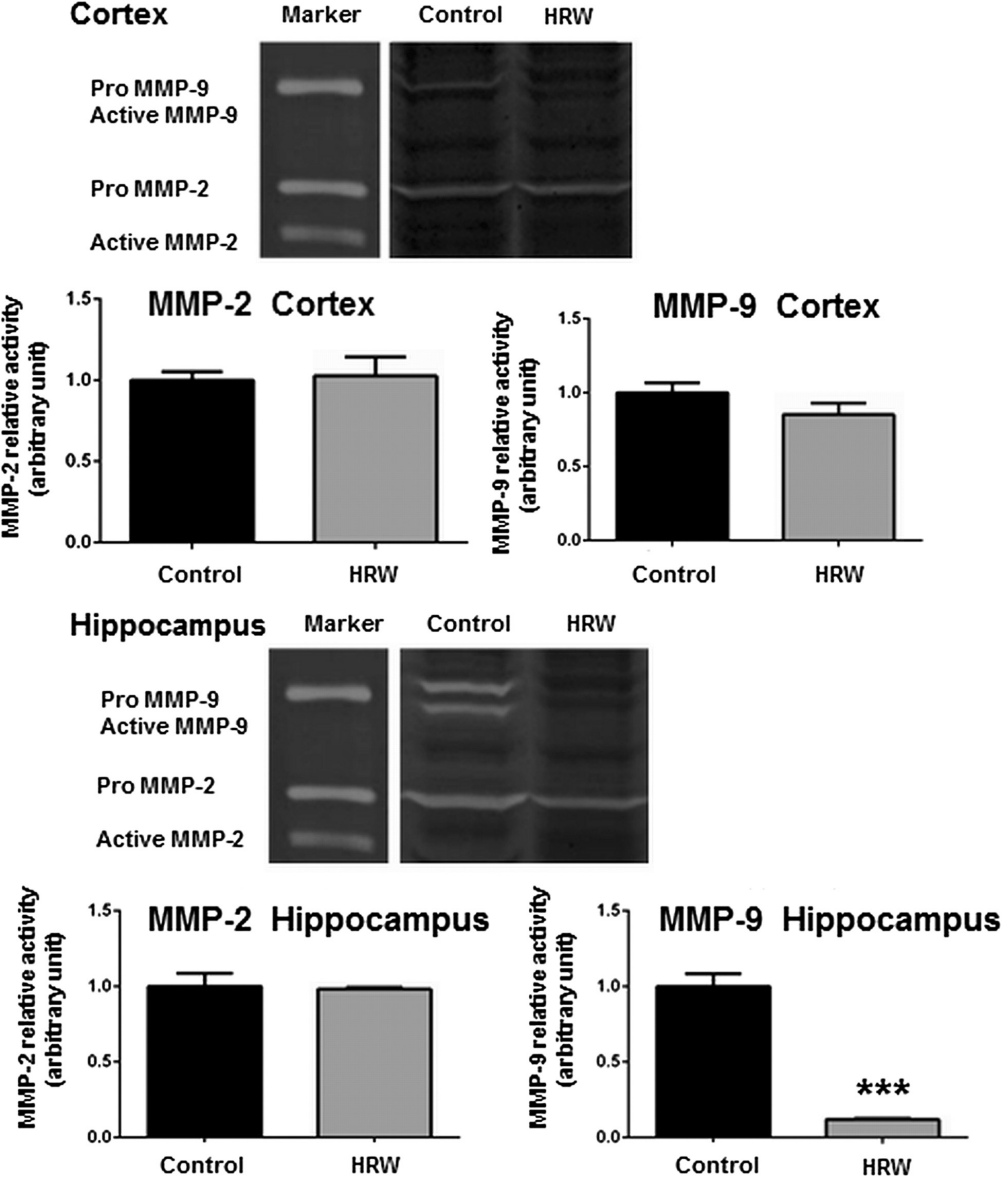
Effect of hydrogen-rich water (HRW) on the activity of matrix metalloproteinases (MMPs) in the brain. Representative images of gelatin zymography and quantification of the relative optical density of MMP-2 and MMP-9. In the cortex, there were no significant differences in MMP-2 and MMP-9 activities (*P* = 0.84 and 0.20, respectively). In the hippocampus, MMP-9 activity was significantly greater in the control group compared to the HRW group (*P* < 0.001), whereas there was no significant difference in MMP-2 activity (*P* = 0.84). Data were analyzed by an unpaired *t*-test. Values are expressed as mean ± SEM. *n* = 6 in each group. ***P < 0.001 vs. control group.

**Figure 9:**
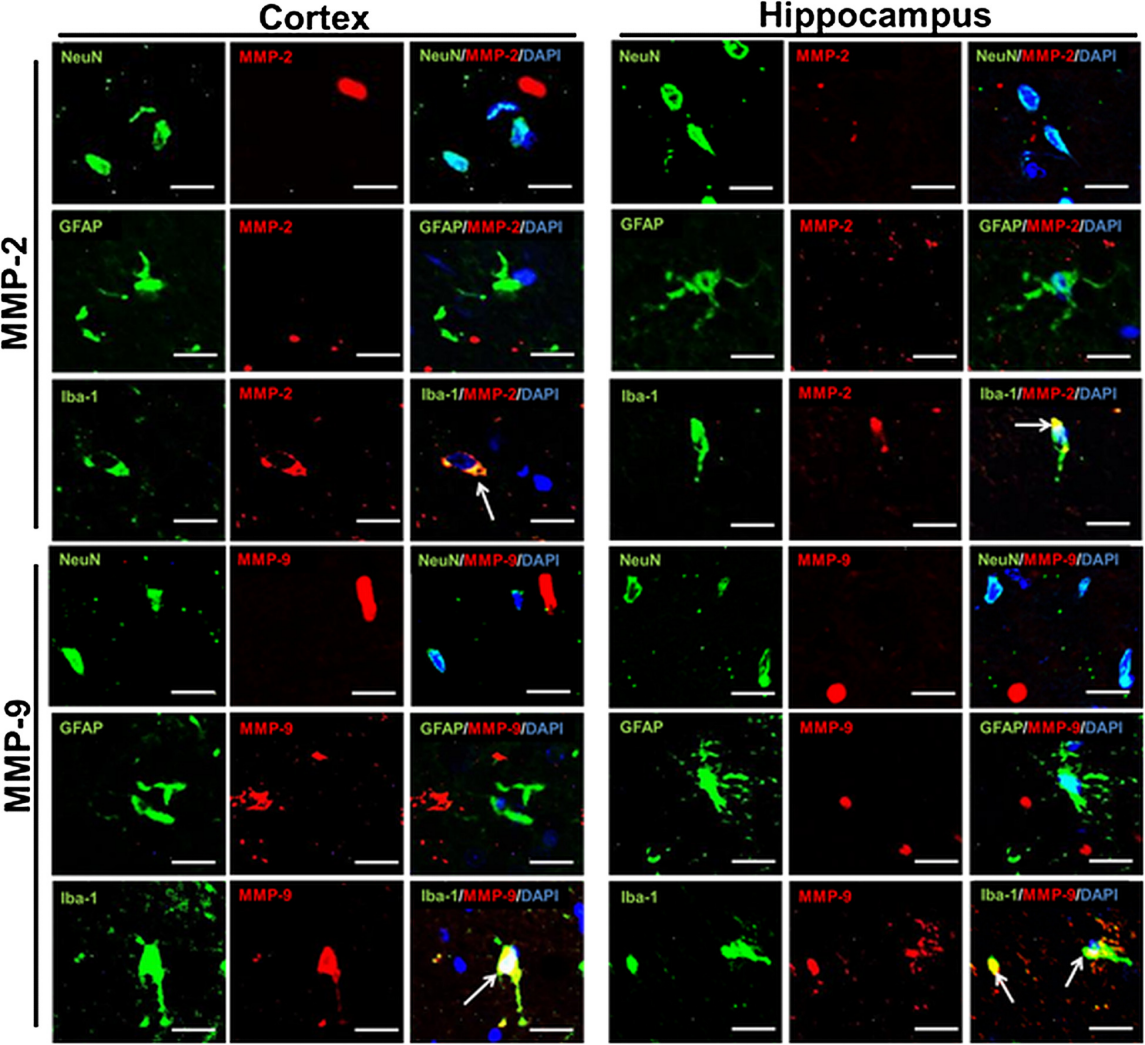
Immunofluorescence staining for matrix metalloproteinases (MMPs). Double staining for neuronal nuclear antigen (NeuN), glial fibrillary acidic protein (GFAP), and Iba-1 (green) showing that MMP-2 and MMP-9 (red) were expressed in the microglia (arrows), but not in neurons and astrocytes (nuclei, 4,6-diamidino-2-phenylindole [DAPI], blue). Co-localization is shown as yellow. Scale bars = 20 μm.

The effect of HRW on MMP activity was assessed by gelatin zymography and immunohistochemistry at 8 weeks after treatment. Zymography showed no significant difference in MMP-2 and MMP-9 activities in the cortex between the two groups (*P* = 0.84 and 0.20, respectively), and that significantly greater MMP-9 activity in the hippocampus in the control group compared to the HRW group (*P* < 0.001) (Figure 8). However, MMP-2 activity in the hippocampus showed no significant difference between the two groups (*P* = 0.84) (Figure 8). Double staining for NeuN, GFAP, and Iba-1 indicated that both MMP-2 and MMP-9 were expressed in the microglia, but not in neurons or astrocytes (Figure 9).

## Discussion

The present study showed that long-term HRW treatment significantly improved neurological function and reduced the prevalence of cerebral lesions in SHRSP, but had no effect on blood pressure. This beneficial effect was associated with (1) reduction in oxidative DNA damage, (2) decrease in BBB leakage in the hippocampus, and (3) suppression of MMP-9 activity in the hippocampus at 8 weeks after treatment (14 weeks of age; before stroke onset).

In the present study, we used 8-OHdG as biomarker for oxidative stress. We observed that 8-OHdG and BBB disruption were detected mainly in the hippocampus, but rarely in the cortex. BBB disruption may be localized in the hippocampus in early life (first 3 months), and subsequently occurs in other regions including the cortex and the basal ganglia as well as the hippocampus in SHRSP [7]. We examined 8-OHdG and BBB function at the age of 14 weeks in SHRSP, or still in early life. Therefore, faint staining of 8-OHdG and weak BBB disruption in the cortex might reflect the low age of the animals. On the other hand, we observed that HRW reduced 8-OHdG and attenuated BBB disruption in the hippocampus, which is consistent with previous reports that BBB disruption in SHRSP is the result of enhanced oxidative stress [8-12]. Further investigations are required to assess the localization of BBB disruption and ROS in the early life of SHRSP.

The present study also investigated the effect of HRW on MMP-2 and −9, which are other important factors in BBB disruption [15-18]. The localization of MMP-2 and MMP-9 in SHR and SHRSP is controversial. Activated MMP-2 and MMP-9 was detected in the cortex and basal ganglia in 6-month-old SHRSP [14]. In contrast, MMP-2 was detected in the cortex but not MMP-9 in non-operated SHR weighing 250–350 g [35] and in non-operated SHR weighing 280–320 g [16]. In this study, MMP-2 was detected in both the cortex and hippocampus, whereas MMP-9 was located mainly in the hippocampus at the age of 14 weeks in the control group. These discrepancies between studies might be due to differences in the age. The present study showed that HRW reduced the MMP-9 activity in the hippocampus. Hydrogen is reported to downregulate many molecules, including MMP-9 and/or MMP-2 [27,28]. Ingestion of HRW decreased oxidative stress and suppressed intimal hyperplasia via reduction in MMP-2 and MMP-9 activation in a rat vein-grafting model [27]. Inhalation of hydrogen gas decreased oxidative stress, exerted neuroprotective effects, and reduced hemorrhagic transformation via reduction in MMP-9 activation in a rat middle cerebral artery occlusion model [28]. Our result is consistent with these previous findings. Therefore, we consider that HRW attenuated BBB disruption through reductions in enhanced oxidative stress and MMP-9 activity in the hippocampus. Further studies are required to investigate whether HRW attenuates MMP-9 activity via reduction in ROS, or independently affects MMP-9 activity and ROS.

The present study showed that 8-OHdG and MMP-2/9 were found in the microglia. Microglia are the resident immune cells in the central nervous system and are essential in the immune response, but are also an important component of the neurovascular unit [36] and therefore are involved in BBB disruption [37]. Recent evidence suggests that microglia can produce ROS [37,38], which is related to BBB disruption. MMP-2 and MMP-9 are produced mainly by the microglia in a rat traumatic brain injury model [37,39]. In addition, the microglia might be related to pathologies in SHRSP [40-42]. Marked proliferation of microglia was observed in the white matter of 20-week-old SHRSP [40]. A marked number of microglia was detected in the white and gray matter at the age of 3 to 5 months [41]. Microglia are activated in the white matter at the age of 28 weeks in SHRSP [42]. Hydrogen has effects on microglia [31,43], which is consistent with our results.

The present study showed that HRW significantly improved neurological function from 9 to 16 weeks after treatment and reduced the prevalence of cerebral lesions in both the cortex and the hippocampus at 16 weeks after treatment in SHRSP. Taken together with the result that HRW attenuated BBB disruption in the hippocampus, these findings support the results of previous studies that BBB disruption occurs in early life and precedes the stroke onset in SHRSP [5,7]. In addition, HRW has beneficial effects in the cortex as well as the hippocampus. In the present study, SHRSPs were given a high salt diet (SP diet), which is often used in this model, resulting in development of very severe hypertension from a young age and short life span (mortality rate of 70% at age 22 weeks in the control group). HRW showed a strong trend toward improvement in overall survival, although not significantly. The cause of death was mainly massive intracerebral hemorrhage in both two groups, suggesting that additional treatment including antihypertensive therapy might be necessary to prevent massive intracerebral hemorrhage and improve overall survival. Further studies are necessary on this point.

The present study has important limitations. We failed to assess whether HRW has the above-mentioned effects in the cortex as well as in the hippocampus in the delayed periods because investigation in the post-stroke period leads to (1) differences in ages (e.g., assessment at the point of stroke onset) or (2) differences in intervals from stroke onset to the assessment point between animals. BBB function, ROS, and MMPs depend on both the age and interval from the stroke onset. Further studies are required to investigate the mechanism of effects of HRW on the cortex in the delayed periods in SHRSP.

## Conclusions

The present study suggests that ingestion of HRW can improve neurological function outcome in the SHRSP model. This beneficial effect may be due to attenuation of BBB disruption via reduction in ROS and suppression of MMP-9 activity in the hippocampus.

## Abbreviations

SHRSP: Spontaneously hypertensive stroke-prone rats
SHR: Spontaneously hypertensive rats
BBB: Blood–brain barrier
MMP: Matrix metalloproteinase
ROS: Reactive oxygen species
8-OHdG: 8-hydroxy-2'-deoxyguanosine
4-HNE: 4-hydroxynonenal
HRW: Hydrogen-rich water
HE: Hematoxylin and eosin
PET: Polyethylene terephthalate
DAPI: 4,6-diamidino-2-phenylindole
NeuN: Meuronal nuclear antigen
GFAP: Glial fibrillary acidic protein
SDS: Sodium dodecyl sulfate
